# Impulsivity and Difficulties in Emotional Regulation as Predictors of Binge-Watching Behaviours

**DOI:** 10.3389/fpsyt.2021.743870

**Published:** 2021-11-10

**Authors:** Jolanta Starosta, Bernadetta Izydorczyk, Katarzyna Sitnik-Warchulska, Sebastian Lizińczyk

**Affiliations:** ^1^Institute of Applied Psychology, Faculty of Management and Social Communication, Jagiellonian University, Kraków, Poland; ^2^Institute of Psychology, Faculty of Philosophy, Jagiellonian University, Kraków, Poland; ^3^SWPS University of Social Sciences and Humanities, Warsaw, Poland

**Keywords:** binge-watching, behavioural addiction, impulsivity, emotional regulation, problematic binge-watching

## Abstract

Recently, the question about the potentially problematic characteristics of binge-watching behaviours has been raised in the contemporary literature. Binge-watching is a highly popular behaviour that involves watching multiple episodes of TV series in one sitting. Studies show that binge-watching can be both an entertaining, but also a potentially problematic, behaviour. Therefore, this research aimed to answer the question about how impulsivity, difficulties in emotional regulation, and one's motivations around why they want to watch a TV series predict problematic binge-watching among a group of Polish young adults. The research group consisted of 645 participants. The following tools were then used to measure the study variables: the Impulsive Behaviour Scale, the Difficulties in Emotion Regulation Scale, the Viewing Motivation Scale, and the Questionnaire of Excessive Binge-watching. Furthermore, a regression analysis was performed on the responses to each measure in order to answer our research questions. Our results show that a lack of premeditation, impulse control difficulties, and having an escapist motivation are all significant predictors of problematic binge-watching behaviours. Furthermore, one's motivations around dealing with loneliness, their motivations around how to best spend their free time, as well as their informative and entertaining motivations were also found to be significant predictors of problematic binge-watching behaviours.

## Introduction

In recent years, there have been significant changes in the ways in which people consume media. The development of streaming platforms such as Netflix, HBO MAX, and Hulu have made it possible for viewers to watch entire seasons of high-quality television (TV) shows in just one sitting (1). Furthermore, these video-on-demand platforms are easily accessible, meaning that people can watch their shows wherever they want—at home, at work, or even on their daily commute (2). Over the last few years, binge-watching has become a highly popular way of spending one's free time. Currently, the extant literature possesses some discrepancies in the definitions of binge-watching, which are related to the amount of episodes watched, the length of the episodes, and the content being consumed (1, 3, 4). However, the most common definition of this behaviour involves watching more than two episodes of a TV show in one sitting (1, 5, 6).

Due to the popularity of binge-watching, questions about its possibly risky or even addictive nature have been raised by various researchers (1, 7–10). Several studies have found that binge-watching can be examined multi-dimensionally (1, 11). For example, it can be both a highly entertaining and healthy way of spending one's free time, but can also be an unhealthy and problematic behaviour (1, 4, 11). Furthermore, some authors state that problematic binge-watching may even have certain similarities to substance or behavioural addictions (4, 10, 12, 13). Prior research has shown that binge-watching is a highly engaging behaviour, meaning that it could lead to an increased loss of control over time (8, 11, 13–15). Some studies have found that participants report experiencing various difficulties in attempting to reduce the time that they spend binge-watching TV series (8, 16). Consequently, excessive binge-watching can potentially negatively affect the life of a given individual; for example, it may lead to a neglect of one's responsibilities or even negative social or health consequences (such as poor sleeping habits or unhealthy eating behaviours) (7, 10, 13, 17–19). As such, it is inferable that problematic forms of binge-watching likely have similar characteristics to other forms of behavioural addictions, such as Gaming Disorder, as mentioned by the WHO in the ICD-11 (20), or with Internet Gaming Disorder, as outlined in section III of the DSM-5 (21). The symptoms of gaming addiction, specifically, consist of impaired control over the behaviour, prioritising gaming over all other activities, as well as a continuation or escalation of gaming activities despite negative consequences (20, 21).

The similarity of problematic binge-watching with other behavioural addictions means that conducting more research into this phenomenon is essential. Research shows that impulsivity is one of the main risk factors for the development and maintenance of both substance and behavioural addictions (22–26). Impulsivity is defined as a tendency to act rapidly without thinking about the consequences (22). People with a high level of impulsivity are often characterised as experiencing difficulties with delaying gratification, as well as focusing too much on the task and being overly persistent. Moreover, they tend to make irrational and unplanned decisions (27). Studies show that this trait is also associated with increased risk-seeking (22). As such, impulsivity is closely related to losing control over one's behaviour, which is one of the main criteria of addictive disorders (20, 21, 28, 29). Impulsivity is also associated with emotional regulation, which is defined as one's ability to alter their own emotional experiences by initiating, maintaining, or modifying the frequency and intensity of the experienced emotions (30). Gratz and Roemer (31), for example, emphasise that emotional regulation consist of an awareness, acceptance, and understanding of one's own emotions, as well as the ability to control impulsive behaviours that stem from having the knowledge on how to act in accordance with the desired outcome when one experiences a negative emotion. It also includes the ability to use appropriate emotional regulation strategies in a given situation. Studies show that individuals with difficulties in regulating negative emotions tend to immediately indulge in their impulses, often needing to weaken the intensity of any negative and unpleasant emotions that they are experiencing (29, 32). Furthermore, research indicates that difficulties in emotional regulation are risk factors for the development of an addiction (32–35). Moreover, people who believe that a substance or behaviour can be used to mitigate their negative emotions, escape reality, as well as help them in coping with stress, anxiety, or sadness, are more likely to develop an addiction of either the substance or the behaviour (24, 32, 36, 37). Therefore, it is important to investigate whether binge-watching is also a way in which people attempt to regulate their emotions and if its excessive usage is associated with one's degree of impulsivity.

The theory of gratification and uses could be used to examine peoples' motivations around engaging in binge-watching behaviours (38). This theory states that people use media, such as the Internet or TV, to satisfy certain goals. The majority of research shows that people tend to binge-watch TV series for entertainment and relaxation (5, 38, 39). On the other hand, authors have also emphasised the roles of escapism and coping with loneliness in peoples' engagement with binge-watching behaviours (11, 39–41). Thus, it becomes clear that people often use binge-watching as a way to seek a sense of positive gratification in order to escape negative emotions. Furthermore, people also binge-watch TV series to enhance their social connexions and to spend time with their friends or families (5). The results reported in recent research indicate that having an informative motivation is another important factor for engaging in binge-watching behaviours (1, 12, 40, 42). Accordingly, it means that people binge-watch to satisfy their cognitive needs. The question then arises about which of these identified motivations is the strongest predictor for the development of the more problematic forms of binge-watching.

Binge-watching is currently a highly popular behaviour, especially during the current global pandemic (43). It is also one of the most common ways of spending one's free time among young adults (44). Furthermore, studies indicate that young adults have a greater tendency to binge-watch on a daily basis. This is notable considering the fact that entering adulthood is often related to an increased risk of substance dependence and behavioural addictions (24, 45, 46). This is likely due to difficulties with adapting to new roles and duties, as well as challenges in making decisions in terms of one's career, education, and relationships (47). Because young adults tend to binge-watch on a daily basis, and are more prone to the development of addictions, it is important to develop a deeper understanding of the psychological conditions underlying problematic binge-watching in this population group.

Thus, this study investigated the psychological predictors of problematic binge-watching, whose definition in the present research was formulated based on the definition of Internet Gaming addiction included in section III of the DSM-5 (21), that of Gaming Disorder as included in the ICD-11 (20), as well as Griffiths model of addiction (48). Based on the clinical classifications of Gaming Disorder and Internet Gaming addiction (7, 20, 21, 48), problematic binge-watching is defined herein as a behaviour whose symptoms consist of a preoccupation with the given behaviour, increasingly prioritising engaging in binge-watching over other activities, an inability to reduce the time one spends on binge-watching, withdrawal symptoms and mood modification, a loss of control over this behaviour, and a continuation of binge-watching despite the occurrence of negative social and health consequences. The conceptualisation of problematic binge-watching, which refers to nosological criteria and the six-component model of addiction, were also applied in the researchers conducted by Orosz et al. (7), Ort et al. (39), and Sun and Chang (49). It is worth mentioning that Forte et al. (9) created a four-factor model of problematic binge-watching, which consists of craving, anticipation, dependency, and avoidance. Furthermore, binge-watching has been defined in various studies as watching more than two episodes of a TV show in one sitting (1, 4–6, 38). Accordingly, the frequency of binge-watching sessions and the average number of episodes watched during each session have been included in various studies when assessing the intensity of binge-watching behaviours (1, 3, 4, 39). Research indicated that the quantity of episodes watched in one binge-watching session and frequency of binge-watching session could be important factors to indicate if the behaviour is problematic or unproblematic (3, 18, 39). It was decided to include abovementioned measures also into this study to capture fuller characteristic of binge-watching. The aim of the study was to find the psychological conditions of problematic binge-watching. The psychological predictors of binge-watching examined in this study include impulsivity, difficulties in emotional regulation, and the different types of motivation underlying one's desire to watch a TV series.

The following research questions were thus proposed:

1. How, and to what extent, does impulsivity predict problematic binge-watching behaviours among Polish young adults?
2. How, and to what extent, do difficulties in emotional regulation predict problematic binge-watching behaviours among Polish young adults?
3. How, and to what extent, do the specific types of motivation underlying one's desire to watch a TV series predict problematic binge-watching behaviours among Polish young adults?

Due to the exploratory character of this research, we decided against drafting specific hypotheses. However, based on the abovementioned studies, it can be assumed that our general hypothesis would be: Impulsivity, difficulties in emotional regulation, and the different types of motivation underlying one's desire to watch a TV series are all significant predictors of problematic binge-watching behaviours among Polish young adults.

## Materials and Methods

### Sample

The study was conducted from September 2020 to January 2021. The invitation to participate in this research project was announced across various Faculties in Jagiellonian University, Silesian Business University, the University of Lower Silesia, the University of Occupational Safety Management, and Silesian University of Technology. Those who indicated an interest to participate were contacted with further information about this research project. Data collection consisted of having participants fill out questionnaires in an individual manner using Microsoft Teams. Participation in this research was completely voluntary and anonymous. No personal data was collected during the research period. Furthermore, the participants were asked to pass on the research invitation to others in their social networks. This study was reviewed and approved by the Ethics Committee of Institute of Applied Psychology, Jagiellonian University in Cracow (nr. 99/2021). The participants provided their written informed consent prior to participating in this study.

The final research group consisted of 752 people. The minimum size of the research sample was determined according to the estimated amount of Polish people aged between 18 and 30 years according to the data from the Central Statistical Office (50). A total of 384 participants was estimated as the minimum sample size necessary. The inclusion criteria were: those aged between 18 and 30 years, those who had declared watching two or more episodes of a TV show in one sitting, and those with an average mental health level (i.e., those without any diagnoses of a mental illness). The exclusion criteria were: never having watched a TV series or have watched <2 episode of a series in a given sitting; being under the age of 18 or over the age of 30 years; having been diagnosed with an affective, psychotic, or anxiety disorder, or with substance dependence; and those who were taking pharmacotherapy or who were currently in psychotherapy as a result of a mental illness. The information about the occurrence of any mental illness and its treatment were collected by a self-report survey. Due to incomplete responses or not meeting the inclusion criteria, 107 people were removed from the analysis. The complete data obtained from 645 participants were then included in our analysis.

### Methods

The Polish version of Whiteside et al. (51). UPPS-P Impulsive Behaviour Scale—originally developed by Ryszard Poprawa (52)—was used to measure impulsivity in this study. This measure consists of 59 items and five subscales, representing five personality facets leading to impulsive behaviour:

- Negative urgency— described as a tendency to engage in impulsive behaviours as a result of negative emotions despite the potentially adverse consequences. Items included: “When I feel rejected, I will often say things that I later regret” and “When I feel bad, I will often do things I later regret in order to make myself feel better now.”
- Positive urgency—defined as a tendency to experience strong impulses to do something in reaction to positive emotional stimulation. Items included: “When I am in a good mood, I tend to lose control” and “When I am very happy, I cannot seem to stop myself from doing things that can have bad consequences.”
- Lack of premeditation—referring to difficulties in planning and evaluating the consequences of a given behaviour before acting. Items included: “Before I enter into a new situation, I like to find out what to expect from it” and “I usually make up my mind through engaging in careful reasoning.”
- Lack of perseverance—described as an inability to focus on and continue with a given task when it becomes frustrating, boring, or difficult. Items included: “I tend to give up easily” and “I finish what I start.”
- Sensation seeking—defined as a tendency to engage in behaviours that are exciting and potentially thrilling. It also includes an openness to potentially hazardous experiences and having a positive reaction by partaking in these kinds of situations. Items included: “I would enjoy driving fast” and “I quite enjoy taking risks.”

The Cronbach's α co-efficients for each of these subscales in this study ranged from 0.83 to 0.94, thereby indicating that this tool possesses a satisfactory psychometric ability. Furthermore, the intercorrelations between the constructs ranged between 0.15 and 0.75 (*p* < 0.001). The study participants ranked their answers on a 4-point Likert scale, including: 1– I totally agree, 2– I partially agree, 3– I partially disagree, 4– I totally disagree.

The Difficulties in Emotion Regulation Scale by Gratz and Roemer (31) was used to measure participants' degree of problems with emotional regulation, which are described as difficulties in modulating, understanding, and accepting one's emotions, as well as an inability to act in a desired/healthy way regardless of one's emotional state. This measure contains 36 items and six subscales.

- Non-Acceptance of Emotional Responses—this reflects the tendency to show negative secondary emotional responses to one's negative affect and an inability to accept one's reactions to experienced distress. Items included: “When I'm upset, I feel guilty for feeling that way” and “When I'm upset, I feel like I am weak.”
- Difficulties Engaging in Goal-Directed Behaviour—defined as experiencing difficulties in focusing on a given task and accomplishing one's goals when an individual experiences negative emotions. Items included: “When I'm upset, I have difficulty focusing on other things” and “When I'm upset, I have difficulty getting work done.”
- Impulse Control Difficulties— referring to difficulties in self-control when one is experiencing negative emotions. Items included: “When I'm upset, I have difficulty controlling my behaviours” and “When I'm upset, I lose control.”
- Lack of Emotional Awareness— defined as difficulties in acknowledging one's own emotions. Items included: “When I'm upset, I take time to figure out what I'm really feeling” and “I care about what I am feeling.”
- Limited Access to Emotion Regulation Strategies— this reflects the tendency to believe that there is nothing that can be done to regulate one's emotions effectively when an individual feels negative emotions. Items included: “When I'm upset, I believe that there is nothing I can do to make myself feel better” and “When I'm upset, I believe that I will remain that way for a long time.”
- Lack of Emotional Clarity—understood as the extent to which an individual understands the emotions that they are experiencing. Items included: “I have difficulty making sense out of my feelings” and “I have no idea how I am feeling.”

The Cronbach's α co-efficients for each of these subscales in this study ranged from 0.80 to 0.85, thereby demonstrating that this measure possesses a satisfactory psychometric ability. Furthermore, the intercorrelations between the constructs ranged between 0.16 and 0.73 (*p* < 0.01). Participants of the study ranked their answers on the 5-point Likert scale, including: 1– almost never, 2– sometimes, 3– about half the time, 4– most of the time, 5– almost always.

The method used to measure peoples' underlying motivations when watching a TV series was the Polish version of the Viewing Motivation Scale (originally created by Alan M. Rubin) as adapted by Starosta et al. (42). The Polish version of this questionnaire consists of 26 items and six scales, as follows:

- Entertainment motivation— wherein an individual watches a TV series to relax and to experience a positive effect. Items included: “Because it amuses me.”
- Motivation to decrease loneliness— an individual watches TV shows to avoid feeling lonely. The TV series and fictional characters then become an individual's company in their solitude. Items included: “So I won't have to be alone.”
- Informative motivation— an individual watches a TV series to learn new information and to gratify their cognitive needs. Items included: “So I can learn how to do things that I haven't done before.”
- Motivation around how to spend one's free time— an individual watches a TV series out of habit and to avoid boredom. Items included: “Because it gives me something to do to occupy my time.”
- Social motivation— an individual watches a TV series to maintain their relationships with others and to spend time together with their friends or family. Items included: “So I can be with other members of my family or friends who are watching.”
- Escapist motivation— an individual watches a TV series to escape from their daily life problems and to avoid negative emotions. Items included: “So I can forget about school or other things.”

The Cronbach's α coefficients for each of these subscales in this study ranged from 0.69 to 0.88. As such, this tool has satisfactory reliability. The intercorrelations between the constructs ranged between 0.09 and 0.43 (*p* < 0.001). Respondents are asked to rate their answers on a 5-point Likert scale, wherein 1— completely untrue, 2— a bit true, 3— very likely, 4— true, 5— definitely true.

The method used to measure problematic binge-watching was the Questionnaire of Excessive Binge-watching created by Starosta et al. (42). This questionnaire was used to examine the intensity of problematic binge-watching among the participating Polish students. This tool's total score can also be used to determine whether one's risk of developing symptoms of excessive binge-watching is low (0–60), medium (61–120), or high (121–180). This method was then used to estimate the levels of problematic binge-watching among our participants, which includes a behaviour whose symptoms consist of a growing preoccupation with it, placing an increasing priority of binge-watching over other activities, a loss of control over the amount of time spent binge-watching (which can lead to a neglect of one's responsibilities), an inability to reduce the time spent on binge-watching, using binge-watching for mood modification, the occurrence of withdrawal symptoms experienced as emotional discomfort (as well as feelings of anger, sadness, or anxiousness when there is limited access to watching TV series), and a continuation of binge-watching despite the occurrence of negative social and health consequences. Items included: “How often do you realise that you spend more time on binge-watching than you have previously planned to?” and “How often do you worry that life without binge-watching would be boring and sad?”.

The Questionnaire of Excessive Binge-watching consists of 30 items. The Cronbach's α coefficients for this and its separate subscales ranged from 0.67 to 0.89, indicating a satisfactory psychometric ability. The intercorrelations between the constructs ranged between 0.26 and 0.61 (*p* < 0.001). Participants ranked their answers on a 6-point Likert scale: 1— never, 2— sporadically, 3— rarely, 4— sometimes, 5— often, 6— always.

The Survey of Binge-watching Behaviour was also used to assess participants' frequency of binge-watching sessions and the amount of episodes that they would watch in each one. The participants were then asked to estimate their average number of binge-watching sessions and the average number of episodes watched in each one.

**Figure 1:**
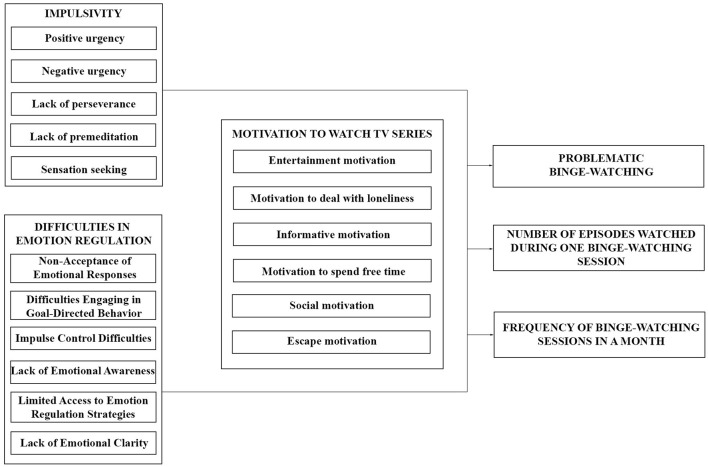
Research model.

The variables measured by these methods are graphically presented in Figure 1. This model was then used to answer the research questions stated in the Introduction.

### Statistical Analyses

The analyses of the study data were conducted using IBM SPSS Statistic software—Statistical Package for the Social Sciences. The first step of the analysis involved measuring the descriptive statistics of the research variables. Subsequently, the correlations between the problematic binge-watching and psychological variables, such as impulsivity, difficulties in emotional regulation, and one's motivations underlying their desire to watch a TV series, were all calculated. Next, a stepwise regression analysis was performed. The variables were successively implemented into the model until the appearance of irrelevant predictors. The aim of this analysis was to determine the strength and directionality of each psychological predictor of problematic binge-watching, as well as the number of binge-watched episodes during each session and the frequency of binge-watching sessions. The significance value of the predictors included in the model was set at *p* < 0.05.

## Results

### Sample Characteristics

A group of 645 participants took part in this study. As shown in Table 1, most of the research group were women (83%). The mean age of the respondents was 20 years (*SD* = 3.04). Furthermore, most of the research group were students only (54%), while some were working as well (32%). The percentage distribution of the disciplines being studied by the participants were as follows: social sciences (49.92%), humanities (26.68%), exact sciences (10.23%), and medical sciences (3.88%). It is worth mentioning that this group is divided in half in terms of the participants' relationship statuses −49% were single and 51% were in a relationship at time of this research. Another important characteristic of the research group is their binge-watching behaviours. Most of the participants reported that that they would often watch two to five episodes during a single binge-watching session. However, the remaining 18.45% of the group answered that they would watch six to 20 episodes in one sitting. The results also revealed that most of the research group preferred to binge-watch at home. Nevertheless, some of the respondents did admit to binge-watching while either travelling (9.61%) or working (2.33%).

**Table 1:** Participant characteristics.

	***n***	**%**
**Gender**		
Woman	532	83
Man	101	15
Transgender man	3	0.5
Unknown	7	1
**Occupation**		
Studying	349	54
Studying and working	209	32
Working	87	14
**Relationship status**		
Single	316	49
In relationship	329	51
**Average number of episodes watched in a single**		
**BW session**		
2–5	529	82
6–20	116	18
**Place of BW session**		
Home	645	100
Work	65	10
Travel	13	2

*BW, binge-watching*.

The next stage of the analysis was to measure the descriptive statistics for all the variables. The results are presented in Table 2.

**Table 2:** Descriptive statistics of research variables.

	**Variables**	***M***	**Min**	**Max**	**SD**
Impulsivity	Negative urgency	28.22	12.00	48.00	6.960
	Positive urgency	21.46	11.00	44.00	6.026
	Lack of premeditation	20.40	10.00	39.00	5.663
	Lack of perseverance	29.49	12.00	48.00	7.941
	Sensation seeking	28.74	16.00	53.00	8.059
Emotional regulation	Non-acceptance of emotional responses	14.96	6.00	30.00	6.313
	Difficulties engaging in goal-directed behaviours	15.23	5.00	25.00	4.655
	Impulse control difficulties	15.66	6.00	30.00	4.659
	Lack of emotional awareness	14.31	6.00	30.00	4.794
	Limited access to emotional regulation strategies	21.98	8.00	40.00	6.938
	Lack of emotional clarity	11.96	5.00	25.00	5.128
Motivation to watch TV series	Entertainment motivation	34.63	9.00	45.00	7.308
	Motivation to deal with loneliness	7.32	3.00	15.00	3.471
	Informative motivation	12.04	5.00	25.00	4.546
	Motivation to spend one's free time	13.01	4.00	28.00	4.271
	Social motivation	5.14	2.00	10.00	2.550
	Escapist Motivation	9.03	3.00	15.00	3.376
Binge-watching	Problematic binge-watching	68.28	30.00	157.00	21.75
	Frequency of BW sessions in a month	5.26	1.00	30.00	5.48
	The number of episodes in one BW session	4.18	2.00	20.00	2.01

*BW, binge-watching; n = 645*.

Based on the obtained descriptive statistics, we found that our research group was characterised by moderate levels of impulsivity and difficulties in emotional regulation. Furthermore, the intensity of their specific motivations underlying their desire to watch a TV series and the intensity of their problematic binge-watching were also average. Participants usually binge-watched five times per month (*SD* = 5.48). The average number of episodes watched during each binge-watching session amounted to four (*SD* = 2.01).

### Predictors of Problematic Binge-Watching

The first stage of studying the relations between the variables was to perform correlation analysis. The results showed the existence of multiple significant relations between dependent and independent variables. Those correlations were especially present between psychological conditions and problematic binge-watching, and number of episodes watched during one binge-watching session. The correlations were mainly positive. Accordingly, obtained results justified the assumptions of the correlative-regressive character of the research model. The next stage of research involved performing a forward stepwise regression analysis to measure the factors associated with the symptoms of problematic binge-watching. The results of the analyses are presented in Table 3.

**Table 3:** Psychological predictors of problematic binge-watching.

**Dependent variable**	**Independent variable**			
Problematic binge-watching	*R*^2^ = 0.389, Adjusted *R*^2^ = 0.389, *p* < 0.001			
	**Predictor**	***β***	***SE***	**95% CI**
	Impulse control difficulties	0.164^***^	0.039	14.70–15.30
	Lack of premeditation	0.110^***^	0.034	19.60–20.40
	Escapist motivation	0.233^***^	0.043	8.77–9.23
	Informative motivation	0.174^***^	0.035	11.70–12.30
	Motivation to spend one's free time	0.087^*^	0.040	12.70–13.30
	Motivation to decrease loneliness	0.112^***^	0.038	6.77–7.23
	Entertainment motivation	0.079^*^	0.039	33.50–34.50
Number of episodes watched in one binge-watching session	*R*^2^ = 0.116, Adjusted *R*^2^ = 0.107, *p* < 0.001			
	**Predictor**	***β***	***SE***	**95% CI**
	Lack of emotional clarity	0.205^***^	0.052	10.60–11.40
	Limited access to emotional regulation strategies	0.113^***^	0.043	13.70–14.30
	Lack of emotional awareness	−0.136^***^	0.051	13.50–14.50
	Impulse control difficulties	0.103^***^	0.052	14.70–15.30
	Entertainment motivation	0.143^***^	0.043	33.50–34.50
Frequency of binge-watching sessions in one month	*R*^2^ = 0.086, adjusted *R*^2^ = 0.081, *p* < 0.001			
	**Predictor**	***β***	***SE***	**95% CI**
	Difficulties engaging in goal-directed behaviour	−0.149^***^	0.039	14.70–15.30
	Entertainment motivation	0.150^***^	0.043	33.50–34.50
	Motivation to spending one's free time	0.133^**^	0.047	12.70–13.30

* *p < 0.05,*

** *p < 0.01,*

*** *p < 0.001, CI, Confidence Interval*.

These results indicate that impulse control difficulties and a lack of premeditation in one's behaviours are significant predictors of problematic binge-watching. This means that the higher the intensity of problematic binge-watching, the larger are a person's difficulties with self-control and engaging in the preplanning of an action prior to acting on a given impulse. The highest β value out of all the motivational predictors was that for the escapist motivation (β = 0.233^***^). These results also revealed that the predictive values of both the informative motivation and the motivation to decrease one's loneliness were significant.

Moreover, the motivation around spending one's free time and the entertainment motivation were also found to have a significant impact on problematic binge-watching. These variables explained 39% of the variance of the dependent variable.

The Lack of Emotional Clarity, Limited Access to Emotion Regulation Strategies, and Impulse Control Difficulties are all significant predictors of how many episodes an individual watches in one sitting. Furthermore, our analysis shows that a Lack of Emotional Awareness is a significant predictor for the number of episodes watched during one binge-watching session. It is worth mentioning that it is the only predictor with coefficients of a negative value (β = −0.103^***^). The entertainment motivation was found to be the only predictor for the amount of episodes watched in one binge-watching session. These predictors were found to explain 11% of the variance in the dependent variable.

The entertainment motivation and the motivation around spending one's free time are both significant predictors of the frequency of binge-watching sessions in a given month. Additionally, Difficulties in Engaging in Goal-Directed Behaviours is the only significant predictor from all the independent variables related to difficulties in emotional regulation. This model explains only 8% of variance in the dependent variable.

It is worth mentioning that motivational predictors were mostly found to have higher β coefficients values than those related to impulsivity and difficulties in emotional regulation.

Furthermore, among all significant predictors only three were present in more than one regression model. Entertainment motivation was significant predictor for all dependent variables. However, its β coefficient had the highest value for Number of watched episodes in one binge-watching session and the lowest value for Problematic binge-watching. The comparative analysis showed that differences between models were statistically significant. It may indicate that such motivation to watch TV series is a more important factor in explaining amount of watched episodes and less relevant in predicting severity of problematic binge-watching. Another predictor which occurred in two regression models was Impulse control difficulties. It is worth mentioning that it has slightly higher β coefficient in case of Problematic binge-watching than Number of watched episodes in one binge-watching session. Motivation to spend one's free time was significant predictor for Problematic binge-watching and Frequency of binge-watching session in 1 month. Its β coefficients were slightly higher in the second regression model. The abovementioned predictors were significant factors in explaining dependent variables. However, the detailed analysis showed insignificant differences between these two models.

## Discussion

Binge-watching is a highly common activity among young adults. This behaviour is not only used for entertainment purposes but also shares several symptoms of certain behavioural addictions, such as Gaming Disorder or Internet Gaming Disorder (9, 10, 20, 21). As such, this study assessed the psychological predictors of problematic binge-watching behaviours (including impulsivity, difficulties in emotional regulation, and one's motivations around their desire to watch a TV series). The results of our regression analysis show that the β coefficients of the motivational factors are slightly higher than those of the participants' personal predispositions, such as their impulsivity or difficulties in emotional regulation.

Our results reveal that a lack of premeditation and impulse control difficulties are both significant predictors for problematic binge-watching. This means that excessive binge-watchers tend to experience difficulties in planning and evaluating their behaviours before acting on a given impulse (11, 53). Based on the results of this study, it is inferable that impulse control difficulties are likely related with the occurrence of problematic binge-watching (11). Our results correspond with those obtained by Steins-Loeber et al. (53). Losing control over the time spent on binge-watching can then be related to a neglect of one's responsibilities, poor academic or work performance, an avoidance of social activities, and various negative health consequences (10, 15, 17, 18, 53, 54). The lack of premeditation underlying problematic binge-watchers can also be related to automatic processes that are results of an impulse/reactive system based on associative learning (55). Similar to other behavioural addictions, the decision to engage in problematic binge-watching is likely automatic and depends on the impulsive/reactive neural system and not on prefrontal-cortex related inhibitory control (55–58). Thus, it can be assumed that a person does not think about the potential negative outcomes of engaging in problematic binge-watching or other excessive behaviours, but rather impulsively engages in this behaviour because of automatic impulsive processing, which could be the result of associative learning, a decrease of inhibitory control, and experiencing a sense of craving (9, 55, 58, 59). Furthermore, it is important to mention that some structural characteristics of streaming platforms and TV series could also affect the automatic decision-making surround binge-watching sessions (6, 13). One of the most important structural factors for losing control over this behaviour could be the autoplay feature of several streaming platforms, wherein the next episode is automatically played several seconds after the previous episode has ended, as well as the use of cliff-hangers by series to keep the audience engaged and invested, meaning that they want to know what happens next (6, 13, 60).

Due to the abovementioned facts, it is important to mention that the results of this research show that the escapist motivation is a significant predictor of problematic binge-watching. Other research also shows that people who excessively binge-watch likely do it to mitigate their negative emotions and to distract themselves from their daily life problems (1, 5, 11, 39, 61, 62). Panda and Pandey (5) emphasise the fact that binge-watching can be used as a form of stress relief. Using problematic forms of binge-watching (or any other form of behavioural addiction) as a mode of escapism and emotional regulation has been examined in prior research (11, 38–41, 61, 63–66). The excessive use of a substance or a behaviour to modify a negative emotional state is one of the most basic pathological mechanisms of substance dependence and behavioural addictions (22, 23, 36, 37, 67–69). Similar to other behavioural addictions, the more an individual tries to escape their negative affect and distract themselves from their daily struggles by engaging in problematic binge-watching, the more intense the symptoms of this behaviour then become (5, 11, 70, 71). Furthermore, Panda and Pandey (5) found that, when individuals feel negative emotions after spending too much time binge-watching, their need to continue binge-watching then increases. As such, it can be assumed that individuals who use binge-watching to escape from their emotions may then lose control over this behaviour as they begin to perceive binge-watching as a priority over their other responsibilities and continue their excessive binge-watching despite the occurrence of negative social and health-related consequences (5, 11, 53). It is worth mentioning that some studies have also outlined the existence of a bidirectional causality between behavioural addictions and affective and anxiety disorders (72–75). Furthermore, some studies have found that excessive binge-watching is also related to the occurrence of anxiety and depression (6, 49). Similar to other behavioural addictions, problematic binge-watching is often used as a temporary relief from an intolerable and unclear emotional state (13, 32, 33, 61, 64, 65).

Another significant factor in explaining problematic binge-watching is peoples' experience of the informative motivation. Research shows that heavy binge-watchers engage in this activity to learn new information and to satisfy their cognitive needs (4, 40). Some researchers have also found that binge-watchers tend to engage in highly immersive and complex narratives, as these provide them with a sense of gratification (40, 76–79). Furthermore, some researchers have reported that heavy binge-watching is also related to the phenomenon known as “fear of missing out” (known colloquially as “FOMO”) (77, 80). Conlin et al. (77) and Anghelcev et al. (80) found that people have a tendency to binge-watch more due to a perceived need to collect information that then helps them to participate in social discourse. Furthermore, Anghelcev et al. (80) outline the fact that heavy binge-watchers seek information because others within their social networks often seek their opinions on related topics. This is similar to the assumption made by Shim and Kim (40) who stated that heavy binge-watchers have a high need for cognitive fulfilment and, as such, are more prone to exploring and forming an increased fascination with a TV show or specific fictional character. This fascination with a character could also be related with another significant predictor— using TV series as a means to deal with loneliness, which is often related to a tendency to create parasocial relationships with fictional characters (39, 81). Studies show that loneliness is yet another negative emotion that people often wish to avoid by engaging in either excessive binge-watching or the problematic use of the Internet, with fictional characters then replacing their lack of social connexions so that a person does not feel as lonely (79, 81, 82). We also found that social motivation is a non-significant predictor of problematic binge-watching. The lack of significance of the social motivation in explaining problematic binge-watching may indicate the fact that people who partake in this behaviour do not do so because of social reasons. Rather, it is likely related with the more isolating characteristics of addictive behaviours (22, 23).

It is also important to mention that the entertainment motivation was the only significant motivational predictor of the number of watched episodes in a given binge-watching session. This implies that watching more episodes is related with maintaining positive emotions and having fun (4, 5, 37, 38, 61), Furthermore, we also found that the entertainment motivation and motivation to use binge-watching to spend one's free time were significant predictors of both problematic binge-watching and the frequency of binge-watching sessions during each month. These findings correspond well with those of prior studies (1, 2, 5, 38, 39, 61). Studies have shown that people often binge-watch because of entertainment reasons (1, 2, 5, 11). Flayelle et al. (11), for example, found that so called “regulated binge-watchers,” who are characterised by low impulsivity and emotional reactivity, may engage in binge-watching because of a sense of eudemonic enjoyment as it gives them a sense of meaningfulness arising from their engagement in the provided media content. Furthermore, Flayelle et al. (11) also emphasised that so called “unregulated binge-watchers,” who are characterised by high impulsivity and emotional reactivity, also engage in excessive binge-watching as a form of entertainment. However, this study found that the latter are more driven by a need to escape negative emotions. Furthermore, it is important to distinguish healthy from potentially problematic binge-watching (1, 2, 11). Similar to other behavioural addictions, as based on the I-PACE model, the risk of developing potentially problematic engagement in binge-watching may depend, not only on motivational factors, but also on other personal predispositions, such as one's temperament, personality traits, psychopathology, genetics, early childhood experiences, affective and cognitive responses to a given stimulus, and their executive functions (22, 55). Brand et al. (55) reported that there is a temporal progression in a person's motivations when developing behavioural addictions. In the early stages, the engagement in the behaviour usually depends on the positive gratification related to the resulting reward anticipation. However, in the later stages of this addiction, the main motivation then becomes a compensatory avoidance of negative emotions (22, 23, 55). As such, there is a need for more longitudinal research investigating the temporal changes in motivation as it occurs in the formation of addictive binge-watching behaviours.

Additionally, we found that impulse control difficulties, limited access to emotional regulation strategies, and a lack of emotional clarity are also significant predictors of the number of binge-watched episodes in a given session. Studies show that people tend to watch more episodes of TV shows because of difficulties in impulse control, as well as from certain structural characteristic of streaming platforms and TV shows, which are often related to both wanting to maintain a positive affect and to escape negative emotions (5, 11, 53). Current research implies that individuals watch many episodes of a given TV show to regulate the emotions that they do not understand nor have the knowledge on how to cope with (5, 11, 53, 70). Consequently, they watch even more TV series to compensate (5). It is important to mention that these difficulties in emotional regulations can be risk factors of various forms of other behavioural addictions as well, such as Internet Gaming Disorder, gambling etc. (32, 33, 36, 37, 53, 63–66).

### Study Limitations

The first limitation of this study was its use of a stratified sampling method. The research group consisted of young adults aged 18–30 years. Subsequently, our results cannot be generalised to the wider population. It is therefore necessary to conduct further research on more age diversified populations to determine if our identified predictors are also significant in explaining problematic binge-watching in other age groups, such as among children, adolescents, middle-aged people, and older adults. The second limitation of this study was the majority of women (*n* = 537) in the research group. Conversely, only 98 men participated in this research. A possible explanation for this is that this study primarily recruited its participants from students of the social sciences and humanities, wherein women are more prevalent than men. For example, according to data given by the Central Statistical Office (83) and the report of the Ministry of Education and Science (84), Polish women constitute most students in these disciplines. Moreover, they also tend to undertake higher education more often than do men. Furthermore, the predominance of women in the research group could also have been due to a higher willingness among this gender group to participate in this research study (85, 86). Additionally, the results of our systematic review reveal the fact that women participate more often in research on binge-watching than do men (1, 4). Perhaps binge-watching, itself, is a more appealing activity for women than men. In future, it will be important to conduct studies that involve a larger population of men in order to confirm our results. The third limitation of this study was that all of its participants were Polish. To generate a more comprehensive understanding of binge-watching behaviours, it is necessary for future studies to conduct comparative research on a more nationally diverse sample to assess if our obtained results can be also generalised to other populations. Another limitation was the fact that we conducted this study during the global COVID-19 pandemic, which would have affected the daily lives of all of the participants. Due to lockdown regulations, individuals were only able to undertake activities at home. As such, engaging more often in binge-watching behaviours could have been the result of a lack of available alternatives (43). The results of the study conducted by Dixit et al. (43) show that boredom and dealing with loneliness were the main motivations underlying peoples' desire to binge-watch during the first year of the global pandemic. Binge-watching has since become a way of coping with stress as a source of immediate gratification. Furthermore, Dixit et al. (43) concluded that using binge-watching as a way to cope with emotional distress may not end when the current pandemic does. As such, this research outlines the risk of developing problematic binge-watching during lockdown that will continue even after lockdown restrictions ease (43). The impact of the pandemic on the results of this research is undeniable. Therefore, it will be necessary to conduct comparative studies following the end of the global pandemic in order to determine whether the conclusions found in this study are still valid in the post-pandemic world. Another limitation of this study concerns its use of self-report methods, wherein one of major weaknesses is related to the occurrence of a socially desired response bias and a difficulty in participants being able to properly assess the intensity of their own behaviours on a Likert scale.

## Conclusions

Due to the ever growing popularity of binge-watching, the question about its addictive characteristics when it becomes excessive has arisen in recent literature. Therefore, this study investigated and outlined the psychological predictors of excessive binge-watching among Polish young adults. The results of our regression analyses reveal that a lack of premeditation, impulse control difficulties, having an escapist motivation, and using binge-watching to deal with loneliness all have significant predictive values in explaining the occurrence of problematic binge-watching. Due to these results, it can be assumed that difficulties in impulse control and using binge-watching as a way to escape from and regulate one's emotions are all risk factors of this behaviour becoming excessive and detrimental. Furthermore, having an informative motivation, a motivation to spend one's free time binge-watching, and an entertainment motivation were also found to be significant predictors of binge-watching behaviours. These results correspond well with findings of other scientists. However, there is still a need to conduct further research, not only to generate a better understanding of these phenomena, but to also distinguish healthy binge-watching from its excessive and potentially unhealthy/addictive form. Furthermore, it is necessary to conduct more research on diverse populations. This comparative analysis could show if other nationalities or age groups can be characterised by the same predictors for problematic binge-watching as participants of this study. Due to the ever-increasing risk of problematic uses of new technologies, generating increased knowledge about behaviours like excessive binge-watching is useful for the creation of more effective preventive, prophylactic, and therapeutic interventions.

## Data Availability Statement

The datasets presented in this study can be found in online repositories. The names of the repository/repositories and accession number(s) can be found below: https://ruj.uj.edu.pl/xmlui/handle/item/279533.

## Ethics Statement

The studies involving human participants were reviewed and approved by Ethics Committee of Institute of Applied Psychology, Jagiellonian University in Cracow (nr. 99/2021). The patients/participants provided their written informed consent to participate in this study.

## Author Contributions

JS: research idea, research design, conceptualisation, literature review, data collection, data interpretation, draught manuscript, visualisation, revision of work, and funding. BI: research design, conceptualisation, project administration, work supervision, and revision of work. KS-W: visualisation, work supervision, and revision of work. SL: data interpretation and revision of work. All authors contributed to the article and approved the submitted version.

## Funding

The proofreading and publishing fee of the publication of this manuscript was provided by the Priority Research Area Society of the Future under the programme Excellence Initiative– Research University at the Jagiellonian University in Krakow.

## Conflict of Interest

The authors declare that the research was conducted in the absence of any commercial or financial relationships that could be construed as a potential conflict of interest.

## Publisher's Note

All claims expressed in this article are solely those of the authors and do not necessarily represent those of their affiliated organizations, or those of the publisher, the editors and the reviewers. Any product that may be evaluated in this article, or claim that may be made by its manufacturer, is not guaranteed or endorsed by the publisher.
